# Modulation of post‐movement beta rebound by contraction force and rate of force development

**DOI:** 10.1002/hbm.23189

**Published:** 2016-04-08

**Authors:** Adam Fry, Karen J. Mullinger, George C. O'Neill, Eleanor L. Barratt, Peter G. Morris, Markus Bauer, Jonathan P. Folland, Matthew J. Brookes

**Affiliations:** ^1^ School of Sport, Exercise and Health Sciences Loughborough University Leicestershire LE11 3TU United Kingdom; ^2^ Sir Peter Mansfield Imaging Centre, School of Physics and Astronomy University of Nottingham, University Park Nottingham NG7 2RD United Kingdom; ^3^ Birmingham University Imaging Centre, School of Psychology, University of Birmingham Birmingham B15 2TT United Kingdom; ^4^ School of Psychology University of Nottingham, University Park Nottingham NG7 2RD United Kingdom

**Keywords:** neural oscillations, sensorimotor cortex, event‐related synchronization, event‐related desynchronization, movement‐related beta decrease, post movement beta rebound, magnetoencephalography, MEG

## Abstract

Movement induced modulation of the beta rhythm is one of the most robust neural oscillatory phenomena in the brain. In the preparation and execution phases of movement, a loss in beta amplitude is observed [movement related beta decrease (MRBD)]. This is followed by a rebound above baseline on movement cessation [post movement beta rebound (PMBR)]. These effects have been measured widely, and recent work suggests that they may have significant importance. Specifically, they have potential to form the basis of biomarkers for disease, and have been used in neuroscience applications ranging from brain computer interfaces to markers of neural plasticity. However, despite the robust nature of both MRBD and PMBR, the phenomena themselves are poorly understood. In this study, we characterise MRBD and PMBR during a carefully controlled isometric wrist flexion paradigm, isolating two fundamental movement parameters; force output, and the rate of force development (RFD). Our results show that neither altered force output nor RFD has a significant effect on MRBD. In contrast, PMBR was altered by both parameters. Higher force output results in greater PMBR amplitude, and greater RFD results in a PMBR which is higher in amplitude and shorter in duration. These findings demonstrate that careful control of movement parameters can systematically change PMBR. Further, for temporally protracted movements, the PMBR can be over 7 s in duration. This means accurate control of movement and judicious selection of paradigm parameters are critical in future clinical and basic neuroscientific studies of sensorimotor beta oscillations. *Hum Brain Mapp 37:2493–2511, 2016*. © **2016 The Authors Human Brain Mapping Published by Wiley Periodicals, Inc**

**Abbreviations**
FRD: False discovery rate
GABA: Gamma aminobutyric acid
MEG: Magnetoencephalography
MNI: Montreal Neurological Institute
MRBD: Movement‐related beta decrease
MVF: Maximum voluntary force
PMBR: Post movement beta rebound
RFD: Rate of force development
SAM: Synthetic aperture magnetometry

## INTRODUCTION

Neural oscillations are a ubiquitous phenomenon generated in multiple brain regions and observable using both invasive recordings such as electrocorticography and scalp based measurements such as magnetoencephalography (MEG). These oscillations comprise periodic signals typically measured in the 1–200 Hz frequency range, and are generated by rhythmic electrical activity synchronised across neurons. They were first reported by Hans Berger [Berger, 1929], who measured differences in electric potential across the scalp and noted the existence of an 8–13 Hz “alpha” rhythm. Further prominent frequency ranges have subsequently been identified including the delta (1–4 Hz), theta (4–8 Hz), beta (13–30 Hz) and gamma (30–200 Hz) bands. Measurable oscillations are present even when the brain is at “rest” and for many years such effects were considered “brain noise”. However more recently it has been shown that oscillations play an important role in co‐ordinating brain activity, with subtle and focal spatiotemporal changes in oscillatory signatures being linked to stimulus presentation [Stevenson et al., 2011], attentional shifts [Bauer et al., 2014] and task performance [Puts et al., 2011].

In the sensorimotor system, motor action has been linked with robust changes in neural oscillations in the beta band (See Cheyne [2013] and Kilavik et al. [2013] for reviews). During preparation and execution of movements, a decrease in beta amplitude is observed, beginning shortly before movement onset and sustained throughout movement, with the largest effect occurring local to contralateral primary sensorimotor cortex [Jasper and Penfield, 1949; Jurkiewicz et al., 2006; Pfurtscheller et al., 2003; Salmelin and Hari, 1994]. This is known as the movement related beta decrease (MRBD). Following movement cessation, beta oscillations exhibit a period of elevated amplitude, known as the post‐movement beta rebound (PMBR), which can be several seconds in duration [Jurkiewicz et al., 2006; Pfurtscheller et al., 1996]. These beta band amplitude changes are extremely robust across individuals, they occur during both internally and externally cued movements [Pfurtscheller and Lopes da Silva, 1999] as well as during cognitive tasks that require a motor component [Brookes et al., 2012]. In addition, similar effects are observed even in the absence of movement if, for example, a subject is asked to ‘think about moving’ [Pfurtscheller et al., 2005; Schnitzler et al., 1997], suggesting that they are not related exclusively to motor output but a more general property of the sensorimotor system.

Despite the robustness, sensorimotor beta modulation remains relatively poorly understood. High amplitude beta oscillations are thought to reflect inhibition [Cassim et al., 2001; Gaetz et al., 2011], a hypothesis supported by quantifiable relationships between beta amplitude and local concentrations of the inhibitory neurotransmitter gamma aminobutyric acid (GABA) [Gaetz et al., 2011; Hall et al., 2011; Jensen et al., 2005; Muthukumaraswamy et al., 2013]). This means that the observed MRBD likely reflects an increase in processing during movement planning and execution. In contrast, the PMBR is thought to reflect the active inhibition of neuronal networks recruited during the preparation and execution phases of motor activity [Alegre et al., 2008; Solis‐Escalante et al., 2012]. What is clear from recent work is that beta modulation, both during and after stimulation, has great potential to be used as a biomarker for pathology, with examples including Parkinson's disease [Hall et al., 2014b] and schizophrenia [Robson et al., in press]. Further, the PMBR has been used in neuroscientific applications ranging from characterization of neural plasticity [Gaetz et al., 2010; Mary et al., 2015] to use in brain computer interfaces [Pfurtscheller and Solis‐Escalante, 2009]. However, despite a vast number of emerging applications, precise characterization of the neural generators of MRBD and PMBR, including their modulation by task, remains incomplete.

Previous studies have shown little modulation of MRBD with task parameters. For example, the reduction in beta amplitude during volitional contractions of the fingers/arm has been shown to be unrelated to movement speed [Stancák and Pfurtscheller, 1995; Stancák and Pfurtscheller, 1996] and the weight of a manipulated load [Pistohl et al., 2012; Stančák et al., 1997]. In agreement, Stevenson et al. [2011] showed that event related decreases in beta amplitude in the visual cortices were not modulated by changing stimulus intensity. Such findings have led some authors to describe event related beta amplitude decrease as a cortical “gate” with a ‘switching off’ of beta oscillations necessary to facilitate local processing. In other words, local neuronal activity supporting information processing is incompatible with sustaining a synchronous resting rhythm. Given local neurons can't do both things at the same time, the rhythm decreases in favour of more complex neural activity [Brookes et al. 2015]. PMBR is more variable in its relationship with task parameters. Stevenson et al. [2012] found the beta rebound to correlate negatively with inter‐stimulus interval during median nerve electrical stimulation. A greater PMBR has also been observed following finger extension movements performed against a heavy resistive load compared to unloaded extensions [Stančák et al., 1997], whereas no systematic difference in PMBR was identified following slow and brisk finger movements [Stančák et al., 1997; Stancák and Pfurtscheller, 1995; Stancák and Pfurtscheller, 1996]. At face value, this may suggest that PMBR is related to the magnitude of force output, but not to the speed of muscular contraction. However, it is important to note that, even during apparently simple movements, numerous parameters are changing simultaneously such as contraction force, rate of force development (RFD), joint position and velocity of movement (these determine the type of contraction, i.e., concentric, isometric or eccentric). To date, precise experiments investigating neuro‐oscillatory behaviour in response to isolated movement parameters are lacking.

In the present paper, we employ an isometric (static) task to remove the influence of movement (joint position, velocity and direction of movement etc.) and thus enable two fundamental parameters of motor output, force and RFD, to be examined in isolation. We employ MEG to measure MRBD and PMBR in experiments where force and RFD are modulated systematically. In this way, we will show that although MRBD is unchanged by force or RFD, PMBR can be modulated systematically, both in amplitude and duration.

## METHODS

### Subjects

Fifteen healthy adults (11 males, 2 left handed, age 28 ± 5 (mean ± std) years) with no known history of neurological conditions or neuromuscular/skeletal disorders took part in the study. The experimental procedures were approved by the Loughborough University Ethical Advisory Committee, and each subject provided written informed consent. All experimental measurements were carried out using the MEG facility at the Sir Peter Mansfield Imaging Centre, University of Nottingham, United Kingdom.

### Experimental Protocol

**Figure 1:**
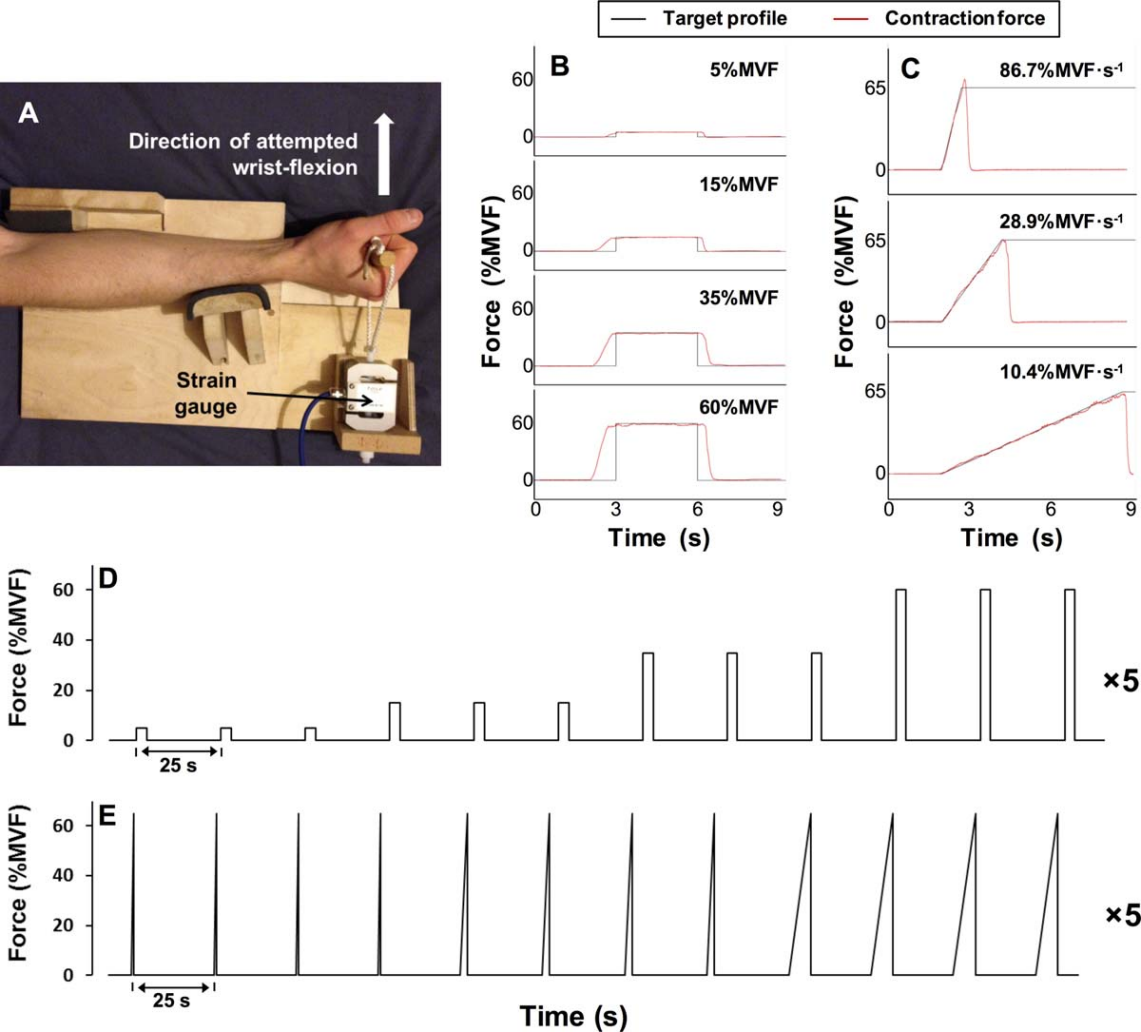
(**A**) A photograph of the isometric wrist‐flexion dynamometer. (**B**/**C**) Each target force profile (black) with single examples of real‐time visual feedback showing contraction force overlaid (red). (**B**) Target profiles for the constant‐force contractions at 5%, 15%, 35% and 60% maximal voluntary force. (**C**) Target profiles for the ramp contractions, with rates of force development of 86.7%, 28.9% and 10.4% maximum voluntary force output per second. (**D**) A schematic diagram of the constant‐force contractions experiment. (**E**) A schematic diagram of the ramp contractions experiment. [Color figure can be viewed in the online issue, which is available at http://wileyonlinelibrary.com.]

Subjects were seated upright in the MEG system with their right forearm and hand positioned in a custom built isometric wrist‐flexion dynamometer that was secured rigidly to the armrest of the MEG system chair. The dynamometer held the subject's forearm in a neutral position of pronation/supination, radial/ulnar deviation and wrist flexion/extension. In all experiments, subjects were asked to exert wrist‐flexion force against a cylindrical handle that was attached in series to a strain gauge (see Fig. 1A). During the experiment, subjects viewed a visual display that showed (in real‐time) force output as a function of time. Subjects were shown a temporal profile of target force output prior to the initiation of contraction and attempted to match their force output to the target profiles. During the contraction, real‐time measured force output was overlaid on the target profile and thus provided visual feedback (see Fig. 1B,C). Two separate experiments were undertaken in a pseudo‐randomised order across subjects:

- ***Experiment 1—Constant‐force contractions***: Subjects performed contractions at four different force levels which were set at 5%, 15%, 35% and 60% of the individual subject's maximum voluntary force output (MVF). Each contraction involved holding the target force as steadily as possible for 3 s. The target profile (see Fig. 1B) appeared on the visual display 3 s prior to the start of the prescribed constant‐force contraction, and remained on screen for a total of 9.25 s. A new target profile appeared every 25 s. Three contractions were performed at each force level, before proceeding to the next force level in ascending order. This sequence of 12 contractions was repeated five times, for a total of 60 contractions (15 contractions at each force level ‐ see Fig. 1D).
- ***Experiment 2—Ramp contractions*:** Subjects performed ramp contractions (i.e., a linear increase in force output over time) at three different rates of force development (RFDs). These rates were set at 86.7%, 28.9% and 10.4% of the subject's maximum voluntary force per second (MVF·s^−1^). Each contraction involved following a target profile that increased linearly from rest to 65%MVF, in a time of 0.75, 2.25 or 6.75 s, respectively (see Fig. 1C). The target profiles appeared 2 s prior to the start of the prescribed ramp contraction and remained on screen for 9.25 s. A new target profile appeared every 25 s. Four contractions were completed at each RFD before proceeding to the next RFD in descending order, with this sequence of 12 contractions repeated five times for a total of 60 contractions; 20 at each RFD (see Fig. 1E).

In all subjects, the complete experimental session comprised determination of MVF, followed by completion of the two MEG experiments described above. To determine a subject's MVF, subjects performed three maximal voluntary contractions (no MEG acquisition), with 30 s rest between each. Subjects were instructed to exert a maximum effort of wrist‐flexion force continuously for 3 s, with visual feedback and verbal encouragement provided. MVF was determined as the overall peak force (averaged over a 200 ms epoch) during these three contractions. In all subjects, a familiarisation session was completed 3–14 days prior to the experimental session. This involved subjects undertaking the constant‐force, ramp and MVF contractions until they were able to perform each task with a high degree of accuracy. During MEG acquisition subjects were instructed to refrain from any movements other than the prescribed wrist‐flexion. No verbal feedback was provided during performance of the experiments. Subjects were asked to abstain from strenuous or atypical exercise for 36 hours prior to the study, and to avoid the intake of nutritional stimulants (e.g. caffeine) within 2 hours of the study.

### Data Collection

MEG data were acquired at a sampling frequency of 600 Hz using a 275 channel CTF MEG system (MISL, Coquitlam, Canada) operating in third order synthetic gradiometer configuration. Three localisation coils were attached to the head as fiducial markers (nasion, left preauricular and right preauricular) prior to the recording. Energising these coils at the start and end of data acquisition enabled localisation of the fiducial markers relative to the MEG sensor geometry as well as determination of total head movement. Note that subjects who moved more than 8 mm between head position measurements were removed from subsequent analyses (with the exception of a small number of individuals where head movement was known to have occurred between the end of the experiment and the measurement of head position).

In order to co‐register brain anatomy to the MEG sensor array, prior to the MEG recording each subject's head shape was digitised relative to the fiducial markers using a 3D digitiser (Polhemus IsoTrack, Colchester, VT). Volumetric anatomical MR images were also acquired using a 3 T MR system (Phillips Achieva, Best, Netherlands) running an MPRAGE sequence (1 mm^3^ resolution). Following data acquisition, the head surface was extracted from the anatomical MR image and coregistered (via surface matching) to the digitised head shape for each subject. This allowed complete coregistration of the MEG sensor array geometry to the brain anatomy, thus facilitating subsequent forward and inverse calculations.

During all experiments, force data were measured using a calibrated S‐beam strain gauge (0–500 N linear range; Force Logic, Swallowfield, UK) housed in the isometric wrist‐flexion rig. Force data were sampled at 2000 Hz using the Spike 2 software package (CED, Cambridge, UK), and an external A/D converter (Micro 1401, CED, Cambridge, UK). A marker was inserted within the MEG and force datasets delineating each individual contraction. This enabled time‐synchronisation between the two data sets. In order to assess the accuracy with which subjects were able to perform the task, deviation between the measured and prescribed contraction force was determined. This was calculated as the mean of the absolute difference between force output and the prescribed force, throughout each contraction.

### MEG Data Analyses

**Figure 2:**
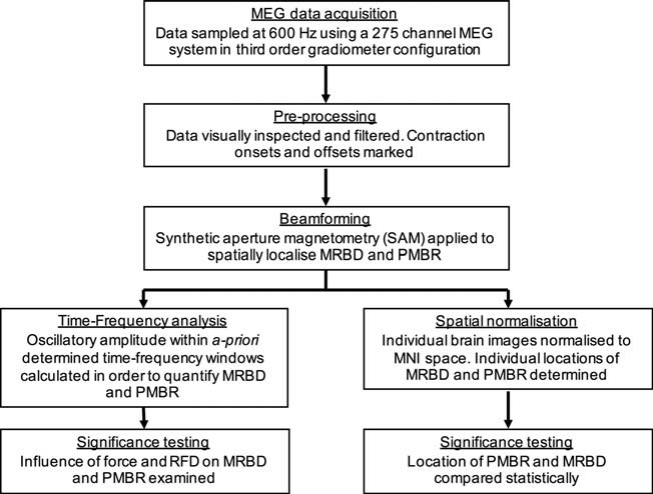
Schematic diagram showing the MEG data analysis pipeline.

An overview of our MEG data analysis pipeline is shown schematically in Figure 2. Initially, MEG data were inspected visually. Common sources of interference, for example the magnetomyogram, magnetooculogram and magnetocardiogram, have well characterised MEG signatures which are easily identified. Here, any trials deemed to contain excessive interference generated via such sources were identified via visual inspection of the MEG data and excluded. Following this, to facilitate consistent analyses across experiments, markers were inserted into the MEG dataset in order to delineate the start and end of a contraction. Contraction onset was defined as the time at which force reached 2% MVF (ramp contractions) or the start of the plateau phase on the target profile (constant‐force contractions); contraction offset was determined as the time at which the contraction force fell below 2% MVF when returning to rest (both experiments).

#### Spatial signature of beta changes

Following pre‐processing, MEG data were analyzed using synthetic aperture magnetometry (SAM) [Vrba and Robinson, 2001], a beamforming variant [van Drongelen et al., 1996; Gross et al., 2001; Hillebrand et al., 2005; Robinson and Vrba, 1998; Van Veen et al., 1997] that has been applied successfully to localise neural oscillatory amplitude changes. Data were first filtered to the beta (15–30 Hz) band. Following this, oscillatory amplitude was contrasted in active and control time windows in order to delineate the spatial signatures of beta amplitude change. To localise MRBD:

- For the constant‐force contractions, the active window was defined as [0.75 < *t* < 2.75 s] relative to contraction onset.
- For the ramp contractions, the active window spanned [0.05 < *t* < 0.65 s], [0.15 < *t* < 1.95 s] and [0.45 < *t* < 5.85 s] relative to contraction onset for the 86.7, 28.9 and 10.4%MVF·s^−1^ contractions respectively.

In order to localise PMBR:

- An active window commencing 0.75 s after contraction offset and lasting for 4 s was used for both the constant‐force and ramp experiments.

In all cases control windows were defined within a [16.8 < *t* < 20.8 s] time window relative to prescribed contraction onset. Note that time windows here necessarily differ to account for paradigm design. However importantly, we ensured that the duration of active and control windows was made equal in all analyses [Brookes et al., 2008]. For beamforming, the forward model was based upon a multiple local sphere head model and the forward calculation by Sarvas [Huang et al., 1999; Sarvas, 1987]. Covariance was computed within the prescribed windows. Pseudo‐t‐statistical images (5 mm^3^ isotropic resolution) were generated showing regions of maximum beta band oscillatory amplitude change between the active and control time windows. Spatial clusters occurring within contralateral sensorimotor regions were identified and used as locations of interest (LOIs) for subsequent analysis.

#### Time‐frequency spectra

Following identification of LOIs, time‐frequency spectrograms were generated for each individual subject in order to measure oscillatory dynamics throughout the experiment. A SAM beamformer was again employed, however here weighting parameters were determined for each LOI using a covariance window spanning the 1–150 Hz frequency range, and a time window encompassing the entire experimental recording (excluding trials rejected for excessive interference). The derived beamformer weights for each location were multiplied by the MEG data (filtered 1–150 Hz) in order to get a “virtual sensor” time‐series showing the evolution of electrical activity at that location. Virtual sensor time‐series were frequency filtered into 31 overlapping frequency bands, and a Hilbert transform was used to generate the amplitude envelope of oscillations within each band. These envelope time‐courses were then averaged across trials within each condition type (i.e., 4 target forces for the constant‐force contractions and three target RFDs for the ramp experiment). Averaged envelopes were then concatenated in the frequency dimension to generate a single time‐frequency spectrum (TFS) per subject, for the LOI specified. TFSs were then averaged over all subjects leaving a single TFS for LOIs at the spatial maxima of the MRBD and PMBR. Note that TFSs were temporally aligned to contraction onset to examine MRBD, and contraction offset to analyse PMBR.

#### The effect of force/RFD on MRBD/PMBR

In order to assess the effect of force and RFD on MRBD and PMBR, first the force measures were analyzed. For the constant‐force contractions, force output was determined in the [0.75 < *t* < 2.75 s] window relative to contraction onset. Average force values were calculated first within each individual (for each force level), and subsequently across individuals to determine an overall group mean. For each ramp contraction, RFD was calculated for each successive 10%MVF increment in force between 2 and 62%MVF (i.e. 10%MVF/time taken). Overall RFD for each individual contraction was then determined as the average RFD of these six increments. Mean averages of these values were again calculated within each individual, and subsequently across subjects.

To assess the effect of force or RFD on neural oscillations, summary values of MRBD and PMBR were extracted from the TFS data in each subject individually. MRBD was calculated as the integral of beta amplitude within the same active time windows as those used for SAM analyses (see above), and was divided by the duration (in seconds) of the window. Thus MRBD represents the mean beta amplitude decrease during contraction. For the PMBR, the integral of the beta envelope was calculated in the [0 < *t* < 10 s] window relative to contraction offset in order to allow for this protracted response to reach baseline. (PMBR was not normalised by duration.) For both the MRBD and PMBR responses, results were generated based on LOIs for each response and each individual separately. This analysis yielded a single value of both PMBR and MRBD, for each condition, for each subject. These values were then averaged across subjects and plotted against either force (constant‐force experiment) or RFD (ramp experiment). In addition, for the beta rebound, we were interested in whether any observable changes in the measured integral were driven by changes in PMBR amplitude or duration. For this reason, post‐hoc tests were also undertaken. Using data averaged across trials and subjects, the PMBR duration was measured as the total continuous time window during which the beta envelope was greater than 20% of its overall maximum value. The PMBR amplitude was estimated as the mean value of the envelope within this window. Note that the 20% threshold was based on a ratio of noise to signal change. The noise level of the envelope timecourses was estimated as 0.3 nAm, based upon the standard deviation of the signal during the rest period (averaged over all conditions and both tasks). The signal was measured as 1.52 nAm (based, conservatively, upon the minimum response across conditions and tasks). The noise level thus represents 20% of the minimum stimulus induced signal change. Thus a temporal window in which the envelope is greater than 20% of the minimum signal represents a continuous robust response. Importantly, the threshold must be defined relative to the signal strength for each condition independently (as distinct from an absolute noise level set at 0.3 nAm) to avoid a confound whereby a larger response amplitude also necessarily generates a larger estimate of response duration.

In order to assess statistically the effect of force and RFD on MRBD and PMBR (integral, duration and average amplitude), permutation testing was employed. A simple linear regression was applied to the graphs of mean MRBD/PMBR versus force/RFD and the gradient of the regression slope was determined. It was reasoned that if force/RFD was affecting MRBD/PMBR then we would observe a non‐zero gradient, whereas under a null hypothesis where force/RFD had no effect, then the gradient would be close to zero. We further reasoned that under this null hypothesis, the order of conditions (i.e. the order of the four force outputs in the constant‐force experiment, or equivalently the order of the three rates of force development in the ramp experiment) could be switched around with no effect on the result. This latter consideration was used to form empirical null distributions testing for the effect of (1) force on MRBD, (2) force on PMBR, (3) RFD on MRBD and (4) RFD on PMBR. In each case, within each subject the order of conditions was permuted randomly (different random permutation for each subject). The data were then averaged across subjects to generate a permuted plot of mean MRBD/PMBR versus force/RFD and the permuted linear regression slope determined. The gradient of the regression slope in the ‘real’ data was then compared to the null distribution, which was generated across 20,000 permutations, and a *P*‐value measured as the integral of the null distribution between the real gradient value and infinity divided by the total integral.

For measurements of MRBD and PMBR integral, a result was considered significant at a level of 0.05; however, this was halved to account for a two tailed test (i.e., either a significantly positive or negative gradient). It was further divided by 4 to account for multiple comparisons across the 4 tests that were undertaken. This meant that a *P*‐value less than 0.00625 in any one single test would indicate statistical significance. Measurements of PMBR duration and amplitude are obviously related directly to integral metrics. These were therefore treated separately to the integral measurements above. Four separate two tailed tests were performed to investigate amplitude and duration in the constant‐force and ramp experiments. In order to account for these multiple comparisons, we applied a false discovery rate (FRD) correction (Benjamini–Hochberg procedure).

#### Location of the MRBD/PMBR response

Finally, we tested for any significant difference in the spatial location of MRBD and PMBR. First, individual brain images were normalised to an anatomical standard [*Montreal Neurological Institute (MNI) brain*] using FLIRT in FSL. Following this, the MNI coordinates for each peak (MRBD and PMBR) in each subject were recorded. Having obtained *x* (left‐right) *y* (anterior‐posterior) and *z* (inferior‐superior) MNI coordinates, the difference in location between MRBD and PMBR was measured as a simple three element vector. It was reasoned that any systematic spatial shift between MRBD and PMBR would manifest as a vector with a consistent direction (e.g., we might hypothesise that in all subjects, PMBR would be shifted in the positive y direction with respect to MRBD). For this reason, significance of the spatial shift was determined using a two‐sided signed rank test of the null hypothesis that any difference in *x*, *y* or *x* coordinates originated from a distribution whose median is zero. The threshold for significance (*P* < 0.05) was Bonferroni corrected (by dividing by 6 to give 0.0083) to account for multiple comparisons across the three elements of the vector, and the two separate experiments (constant‐force and RFD).

## RESULTS

All subjects were able to perform the prescribed tasks. The mean force outputs (mean across subjects ± standard deviation) during the constant‐force contractions were 5.4 ± 0.3, 15.3 ± 0.5, 34.9 ± 0.5 and 59.4 ± 0.5%MVF. The mean RFDs during the ramp contractions were 86 ± 10, 29.7 ± 1.5 and 9.9 ± 0.3%MVF·s^−1^. The accuracy of performed contractions varied with both force and RFD. Mean absolute error increased with prescribed contraction force; 0.5 ± 0.3, 0.6 ± 0.2, 0.8 ± 0.2 and 1.2 ± 0.4%MVF (for 5, 15, 35 and 60%MVF conditions, respectively; *P* < 0.001). Note that the opposite effect was observed when considering this error as a percentage of the prescribed force; 10 ± 5%, 4 ± 1%, 2 ± 1% and 2 ± 1% (*P* < 0.001). Similarly, mean absolute error increased with prescribed RFD during the ramp contractions; 1.8 ± 0.4, 3.1 ± 0.7 and 6.6 ± 1.8%MVF (for 9.6, 28.9, and 86.7%MVF·s^−1^ conditions, respectively; *P* < 0.001). Again this was measured as a decrease when considering percentage error; 18 ± 5%, 11 ± 3% and 8 ± 2% (*P* < 0.001). Correlation between accuracy and the electrophysiological effects of interest (MRBD and PMBR) was also assessed; results of this analysis can be found in the appendix. The mean measured head movement during data acquisition was 4 mm for the force experiment, and 6 mm for the RFD experiment.

**Figure 3:**
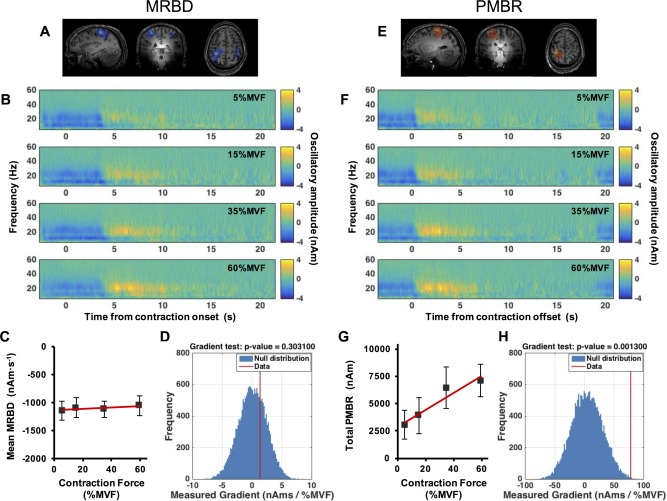
Results of the constant‐force experiment. (**A/E**) Spatial signatures of MRBD (**A**) and PMBR (**E**) in a single subject. (**B/F**) Time‐frequency spectrograms extracted from locations of interest at the peak MRBD (**B**) and PMBR (**F**); upper to lower panels represent (prescribed) 5%MVF, 15%MVF, 35%MVF and 60%MVF contractions. Note that for MRBD, time zero indicates contraction onset; for PMBR, time zero indicates contraction offset. (**C/G**) Mean MRBD during the contraction (**C**) and total PMBR integral over the 10 s post contraction period (**G**) plotted against force. (**D/H**) The null distribution (blue) with the measured MRBD (**D**) and PMBR (**H**) gradient from real data overlaid in red. Note significant (p_corrected_ < 0.05) modulation of PMBR with force output was observed. [Color figure can be viewed in the online issue, which is available at http://wileyonlinelibrary.com.]

Figure 3 shows the primary results of our constant‐force experiment. The spatial signatures of MRBD and PMBR, in an individual representative subject, are shown in Figure 3A,E respectively. A clear contralateral MRBD, local to sensorimotor cortex was observed in all subjects, and a clear PMBR, again local to contralateral sensorimotor cortex was observed in 13 of the 15 subjects (see also the spatial analysis below). MRBD in ipsilateral cortex was also observed for 10/15 subjects, but ipsilateral PMBR was not reliably measured. Time‐frequency spectrograms, averaged across subjects, are shown in Figure 3B,F; Figure 3B shows the case for LOI at the peak MRBD whilst Figure 3F shows the case for LOI at the peak PMBR. In both cases, the upper panel shows 5%MVF, the upper middle 15%MVF, the lower middle 35%MVF and the bottom panel 60%MVF. In all of the TFS plots, blue represents a decrease in oscillatory amplitude with respect to baseline whereas yellow reflects an increase. Note that for MRBD, time zero indicates contraction onset; for PMBR, time zero indicates contraction offset. A clear decrease in beta oscillations (the MRBD) is observed both preceding and throughout the motor task. This is followed by an increase above baseline following task cessation (the PMBR). Given the close spatial proximity of the peaks in MRBD and PMBR it is unsurprising that Figure 3B,F are similar, with the main features observable at both spatial locations. Note also that, in addition to beta band effects, a decrease in mu rhythm (8–13 Hz) is also apparent both preceding and throughout movement, however this was inconsistent across subjects and therefore was not analyzed further. Interestingly, the TFSs suggest that whilst force output appears to have little effect on MRBD, a clear increase in PMBR amplitude with force output is evident.

Figure 3C shows that mean MRBD amplitude remains approximately equivalent for each force output, suggesting that there is no consistent effect of force output on MRBD. Figure 3D illustrates the null distribution (in blue) with the measured gradient from the real data overlaid in red, and shows that that no significant effect of force on MRBD was observed. In contrast, Figure 3G shows the total integral of PMBR plotted against force, with a clear monotonic change in PMBR with force output. Figure 3H illustrates the null distribution with the measured gradient overlaid, and shows that the linear modulation is significant even after correction for multiple comparisons (*P* = 0.0013).

**Figure 4:**
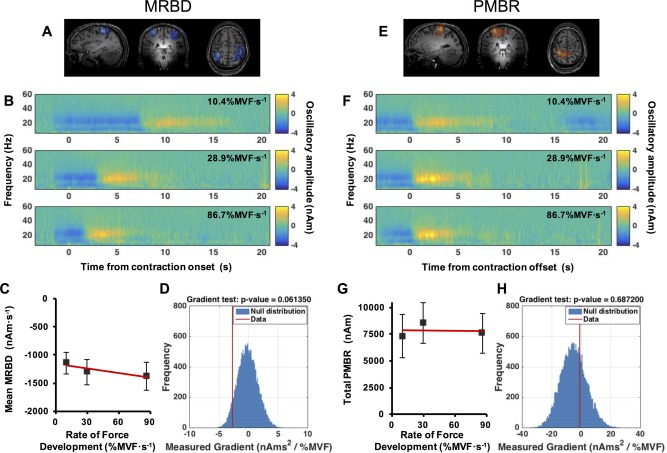
Results of the ramp experiment. (**A**/**E**) Spatial signatures of MRBD (**A**) and PMBR (**E**) in a single subject. (**B**/**F**) Time‐frequency spectrograms extracted from locations of interest at the peak MRBD (**B**) and PMBR (**F**); upper to lower panels represent (prescribed) 10.4%MVF·s^−1^, 28.9%MVF·s^−1^, and 86.7%MVF·s^−1^ contractions. Note that for MRBD, time zero indicates contraction onset; for PMBR, time zero indicates contraction offset. (**C/G**) Mean MRBD during the contraction (**C**) and total PMBR integral over the 10 s post contraction period (**G**) plotted against RFD. (**D/H**) The null distribution (blue) with the measured MRBD (**D**) and PMBR (**H**) gradient from real data overlaid in red. Note no significant modulation of either MRBD or PMBR with RFD. [Color figure can be viewed in the online issue, which is available at http://wileyonlinelibrary.com.]

Figure 4 shows the primary results of the RFD experiment. The layout is equivalent to that of Figure 3. The spatial signatures of MRBD and PMBR, in an individual representative subject, are shown in Figure 4A,E respectively. Contralateral MRBD and PMBR were again observed reliably; ipsilateral MRBD was seen in 12 of 15 subjects whilst no reliable ipsilateral PMBR was measured. Average time‐frequency spectrograms extracted from LOI at the peaks of MRBD and PMBR are shown in Figure 4B,F respectively. A clear decrease in beta oscillations preceding and throughout movement, and a beta rebound occurring on movement cessation is again illustrated. No statistically significant modulation of either MRBD, or PMBR was observed with changing RFD; as illustrated in Figure 4C,D,G,H.

**Figure 5:**
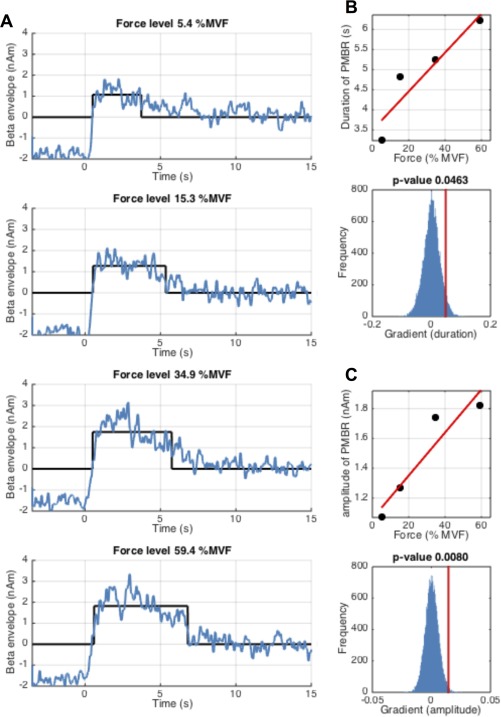
Measurement of PMBR amplitude and duration during the constant‐force experiment. (**A**) Shows the average beta band envelope time‐courses (blue) for each of the four force outputs; time t = 0 denotes contraction offset. The black lines show the estimated duration and mean amplitude of the PMBR. (**B**) Duration of PMBR plotted against force output (upper panel) and the measured gradient (PMBR duration against force) (red line) alongside the null distribution in blue (lower panel). (**C**) Mean amplitude of PMBR plotted against force output (upper panel) and the measured gradient (PMBR amplitude against force) (red line) alongside the null distribution in blue (lower panel). Note that whilst amplitude of PMBR increases significantly with force output, duration fails to reach significance following FDR correction. [Color figure can be viewed in the online issue, which is available at http://wileyonlinelibrary.com.]

Results above show only mean MRBD and total PMBR integral. However, for the PMBR, an increased integral, which recall was measured in the [0 < *t* < 10 s] window post contraction offset, could be driven either by an increase in amplitude of the response, an increased duration of the response, or a combination of the two. For this reason, both PMBR amplitude and duration were tested further. Figure 5 shows PMBR amplitude and duration measured during the constant‐force experiment. Figure 5A shows the time‐courses of the beta envelopes in blue, with estimated duration and mean amplitude overlaid in black. Figure 5B shows the duration of the PMBR plotted against force output (upper panel), and Figure 5C shows amplitude of the PMBR plotted against force output (upper panel). These relationships are tested statistically in the lower panels of Figure 5B,C. Note that the increase in PMBR amplitude with force is significant (*P* = 0.008). However, the apparent increase in PMBR duration with force did not reach significance.

**Figure 6:**
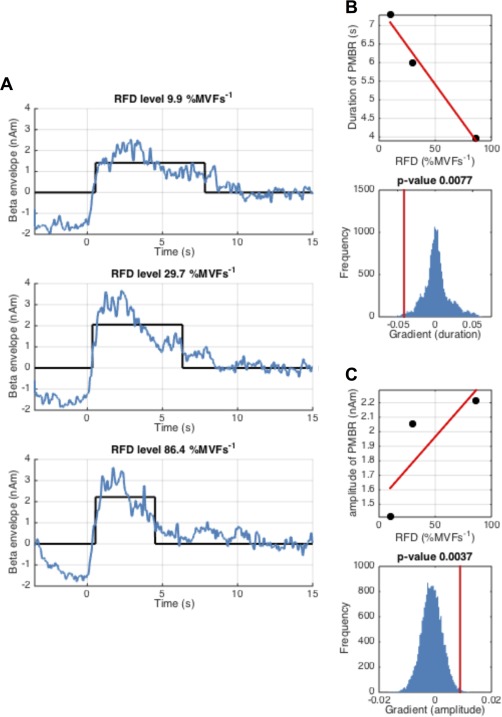
Measurement of PMBR amplitude and duration during the ramp experiment. (**A**) Shows the average beta band envelope time‐courses (blue) for each of the three RFDs; time t = 0 denotes contraction offset. The black lines show the estimated duration and mean amplitude of the PMBR. (**B**) Duration of PMBR plotted against RFD (upper panel) and the measured gradient (PMBR duration against RFD) (red line) alongside the null distribution in blue (lower panel). (**C**) Mean amplitude of PMBR plotted against RFD (upper panel) and the measured gradient (PMBR amplitude against force) (red line) alongside the null distribution in blue (lower panel). Note that as RFD is increased (and the duration of the ramp contraction decreased) the duration of the PMBR is significantly reduced, and PMBR amplitude is significantly increased. [Color figure can be viewed in the online issue, which is available at http://wileyonlinelibrary.com.]

Figure 6 shows the PMBR amplitude and duration measured during the ramp experiment. Figure 6A shows the time‐courses of the beta envelopes in blue, with the estimated duration and mean amplitude overlaid in black. Figure 6B shows the duration of PMBR plotted against RFD whilst Figure 6C shows amplitude of the PMBR plotted against RFD. The lower panels of Figure 6B,C show associated statistical testing. Interestingly, PMBR duration reduces significantly (*P* = 0.008) with RFD whilst PMBR amplitude increases (albeit non‐linearly) significantly (*P* = 0.004). These two significant results combine to generate the negative result (shown in Fig. 4G) that PMBR integral is unaffected by RFD.

**Figure 7:**
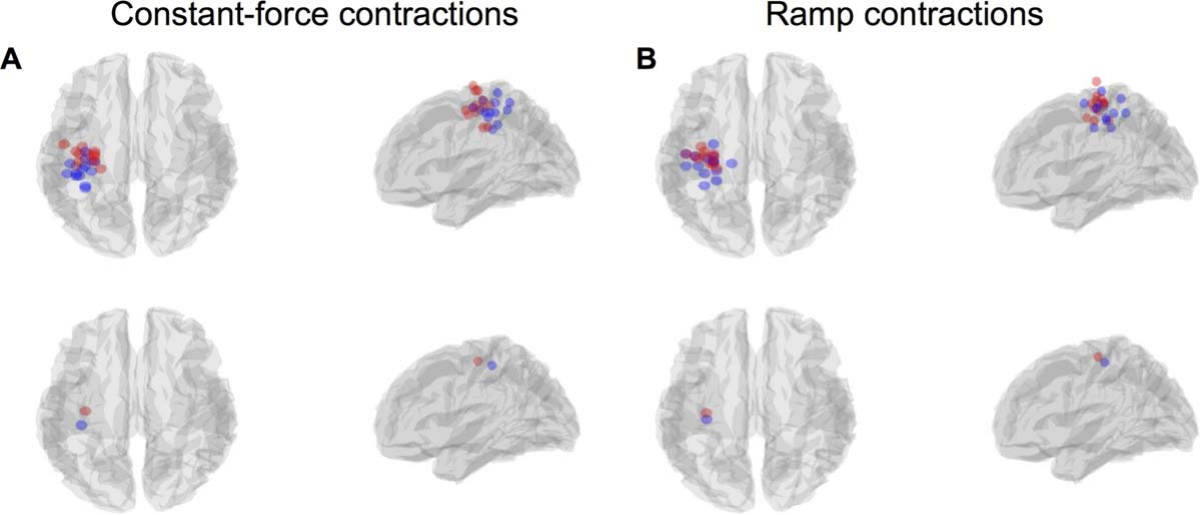
(**A/B**) Peak locations of the MRBD (blue) and PMBR (red) for each individual subject (top row) and the group average (bottom row), in both the constant‐force (**A**) and ramp (**B**) experiments. [Color figure can be viewed in the online issue, which is available at http://wileyonlinelibrary.com.]

Finally, Figure 7 and Table 1 show the results of our spatial analyses, testing a hypothesis that the contralateral MRBD and PMBR are generated in different cortical regions. Figure 7A,B show the localisation for the constant‐force and ramp contractions respectively. The top row of Figure 7A,B show the spatial locations of the derived peaks in all subjects, plotted in MNI space. The bottom rows show group average locations. The mean and standard deviation of the x, y and z coordinates across all subjects are shown in Table 1, alongside the most likely cortical locations of these mean MNI coordinates according to the Oxford‐Harvard brain atlas. The statistical analysis showed that for the constant‐force contractions there is a significant (*p*_corrected_ < 0.05) shift in the anterior (positive y) direction for the PMBR compared to the MRBD. Although the same trend was observed in the ramp contractions this failed to reach statistical significance.

**Table 1:** MNI coordinates and associated most likely cortical locations (according to the Oxford‐Harvard atlas) of the MRBD and PMBR

	MNI coordinates			Cortical location
	x	y	z	
**Constant‐force contractions**				
MRBD	−36.4 ± 1.3	−31.5 ± 2.1	50.7 ± 2.1	Postcentral gyrus
PMBR	−33.7 ± 1.9	−19.9 ± 1.6*	54.9 ± 2.9	Precentral gyrus
**Ramp contractions**				
MRBD	−32.9 ± 2.1	−27.2 ± 2.4	52.7 ± 2.3	Postcentral gyrus
PMBR	−32.6 ± 1.7	−21.4 ± 1.4	58.5 ± 2.5	Precentral gyrus

The * indicates a significant difference from the corresponding MRBD coordinate following Bonferroni correction for the six comparisons made (*P*_corrected_ < 0.05).

## DISCUSSION

Movement induced modulation of neural oscillations in the beta band is one of the most robust neural oscillatory phenomena in the brain. Specifically, in the preparation and execution phases of a motor task, a loss in beta oscillatory amplitude is observed (movement‐related beta decrease, MRBD) and this is followed by a rebound above baseline (post‐movement beta rebound, PMBR) on task cessation. However, despite the robust nature and the clear clinical and neuroscientific value of beta measurements, the phenomena themselves are poorly understood. In this study, we have employed a carefully controlled isometric wrist flexion paradigm to isolate two important movement parameters; namely force output and the RFD. Our results show that PMBR was altered systematically by both parameters; specifically, higher force output results in significantly greater PMBR. A greater RFD results in a PMBR of shorter duration but higher amplitude. In contrast, neither force output nor RFD has any effect on MRBD. These results suggest that PMBR and MRBD reflect functionally separate mechanisms and, consistent with this idea, they were also found to be localised in significantly different cortical areas. These results provide interesting perspectives regarding the interactions between inhibitory and excitatory processes and inspire distinct testable hypotheses.

Our finding that MRBD is not related significantly to either force output or RFD is not surprising, and supports a number of previous studies suggesting that event related beta decrease acts as a cortical “gate,” the magnitude of which is unrelated to stimulus parameters. Our results are in accordance with previous investigations suggesting that neither contraction force [Cremoux et al., 2013; Stančák et al., 1997] nor movement velocity [Stancák and Pfurtscheller, 1995; Stancák and Pfurtscheller, 1996] influence MRBD observed during contractions. In addition, our own previous work has shown a distinct “on/off” property to stimulus evoked beta amplitude reductions, not only in sensorimotor cortex but also in the visual and somatosensory systems [Stevenson et al., 2011; Stevenson et al., 2012]. Likewise, Bauer et al. [2006] has shown that spatial tactile attention has strong effects on the sensorimotor beta rebound, but not on the stimulation induced beta‐suppression, entirely consistent with the pattern of results observed here. There are a number studies which report that beta‐suppression can be elicited even in the absence of direct force output. For example, beta power loss is observed in motor planning [Brookes et al., 2012; Liddle et al., 2016; Pastötter et al., 2012; Tzagarakis et al., 2010; Van Wijk et al., 2009] and motor imagery [Pfurtscheller et al., 2005; Schnitzler et al., 1997]. Given that beta MRBD can occur with no movement, it is perhaps unsurprising that MRBD itself does not modulate with force or RFD. Finally, it is noteworthy that we undertook post‐hoc analyses to test whether MRBD was significantly correlated with the accuracy at which individual subjects performed the task (see appendix for details). Results showed a weak trend that subjects with higher accuracy are better able to supress their beta rhythm during force output (see Fig. 1A). However this correlation failed to reach statistical significance following multiple comparison correction, and was only observed in the constant‐force task (see Fig. 1B). Nevertheless future investigations may further probe this relationship.

In contrast, previous investigation into the PMBR has found it to be more variable across stimulus conditions [Stevenson et al., 2012]. To our knowledge, our current work represents the first demonstration of monotonic increase in PMBR integral with force output, and the first demonstration of increased PMBR amplitude and decreased duration with RFD. Our results are in partial agreement with those of Stančák et al. [1997] who demonstrated a greater PMBR following loaded finger extensions against their heaviest external load compared to their unloaded extensions; although other movement parameters, including RFD, would also have varied between conditions. Contrary to our findings, one previous investigation found no difference in PMBR following isometric elbow flexions between 25 and 75% maximal force [Cremoux et al., 2013]. The reason for this discrepancy likely relates to experimental design. As shown clearly in Figures 3F and 4F, the post movement beta rebound is a protracted response lasting up to 7 s following contraction cessation. Cremoux et al. analyzed PMBR within a <2 s time window, following their constant‐force contractions. Further, their baseline oscillatory amplitude was measured in the −0.4 s to −0.1 s window relative to contraction onset; a relatively short inter‐stimulus‐interval means that this is less than 5.6 s following offset of the previous contraction. This means that baseline was likely computed before the PMBR returned to zero, thus overestimating baseline oscillatory power and MRBD, and underestimating PMBR. This may be a potential reason why Cremoux also observed a slight (albeit not significant) increase in the magnitude of MRBD with increasing force. Although our findings show that PMBR is modulated by both force and RFD, it should be made clear that these are unlikely to reflect the only parameters upon which PMBR depends. Similar to MRBD, the PMBR is also observed in the absence of actual movement (for example PMBR occurs following movement planning but in the absence of an actual movement [Liddle et al., 2016]. It follows therefore that PMBR is a complex signal feature modulated by cognitive processes as well as sensory input and movement parameters.

One argument for the role of beta oscillations is that they are thought to exert an inhibitory influence within the sensorimotor system, with a decrease in beta amplitude potentially reflecting a switch to a state in which a greater range of movements can be made. It has been shown that voluntary movements are slowed during periods of high beta oscillations [Gilbertson et al., 2005], and when beta rhythms are entrained using transcranial alternating‐current stimulation [Pogosyan et al., 2009]. In addition, it is known that attending to a particular location in the body causes shifts in beta amplitude [Bauer et al., 2012; van Ede et al., 2014], consistent with the notion that such events inhibit ipsilateral cortex and promote encoding in contralateral cortex. The transient increase in beta amplitude following a movement (the PMBR) has been suggested to reflect inhibition of motor activity which may facilitate motor control by preventing the generation of further unwanted movements. Some evidence for this theory comes from findings that the PMBR is reduced [Gaetz et al., 2010] and exhibits different morphology [Cheyne et al., 2014] in young children, and increases throughout development [Gaetz et al., 2010]. Though speculative, such findings potentially reflect the fact that young children find fine motor control challenging, and such control develops throughout adolescence. The precise relationship between oscillatory amplitude (which reflects high synchrony across many neurons) and inhibition is not clear, however it has been argued that reduced synchrony allows greater flexibility to encode information in cellular assemblies [Brookes et al., 2015]. It seems intuitive that increased inhibitory control would be required following high force, compared to low force outputs and so the inhibitory hypothesis on the role of beta oscillations would fit with the PMBR modulation shown in the present paper.

Further evidence for the inhibitory influence of beta synchrony comes from neurochemistry. In general, neural oscillations likely depend upon a delicate balance of excitatory and inhibitory neurotransmission. This in turn depends (in part) on glutamate (the major excitatory neurotransmitter in the cortex) and GABA (the major inhibitory neurotransmitter in the cortex). There is some evidence (also reviewed in Kilavik et al., [2013]) to suggest that beta modulation is related to GABAergic inhibition. Jensen et al [2005] enhanced GABAergic currents by administering Benzodiazepines in human volunteers and, using MEG, measured enhanced sensorimotor beta‐oscillations. In agreement with this GABAergic link, Muthukumaraswamy and colleagues [2013] provided evidence that blocking GABA uptake via administration of Tiagabine alters baseline and task induced modulation of beta oscillations. A separate pharmaco‐MEG study by Hall et al. [2011] used administration of the GABA‐A receptor modulator diazepam to show that MRBD is a GABA‐A mediated process whereas PMBR appeared to be generated by a non‐GABA‐A mediated process. Furthermore, Gaetz et al. [2011] have shown, using magnetic resonance spectroscopy, that the magnitude of PMBR across subjects correlates positively with individual subject GABA levels, pointing further towards a positive influence of GABA on PMBR. Collectively, this evidence points towards the beta band response being a potential marker of GABAergic inhibition. If this is indeed the case, then the beta band response offers a direct and non‐invasive way to probe neurochemical imbalance in diseases such as schizophrenia where there is evidence that GABA levels may be disturbed. Indeed recent work demonstrates differences in PMBR in Schizophrenia patients [Robson et al., in press].

One potential further biochemical explanation for the beta rebound is grounded in a phenomenon called adaptation or after‐hyperpolarization currents [McCormick et al., 1993]. These currents, typically involve enhanced efflux of potassium after sustained period of firing causing hyperpolarization of the membrane and therefore an enhanced firing threshold. Hence, even without active top down inhibition, it is conceivable that, due to these adaptation currents and the ensuing shift in the balance between excitation and inhibition, beta‐oscillations become more dominant. The fact that beta‐oscillations can emerge due to an increase in afterhyperpolarization/potassium currents has been shown using in vitro recordings [Kopell et al., 2000; Traub et al., 1999]. The increase of the beta‐rebound with higher levels of force may then be due to an enhancement of these currents, given higher levels of activity. The precise contingency of the beta‐rebound on force, RFD and force duration might then be a function of adaptation currents on the intensity and duration of previous firing in the motor neurons. To test this hypothesis, apart from invasive studies that might allow more direct investigation, neurochemical studies may be useful as, for instance, various neurochemicals such as acetylcholine are known to reduce adaptation currents and allow neurons to fire for sustained periods. One would therefore expect that higher levels of acetylcholine would reduce the beta‐rebound if the outlined hypothesis had any ground. An alternative, although not mutually exclusive, hypothesis would suggest that the PMBR reflected an adaptive functional process, potentially controlled by top‐down inputs. That top‐down processes play a role here, and can diminish specifically the beta‐rebound in a topographical manner has been shown by previous studies [Bauer et al., 2006; van Ede et al., 2014]. In order to understand the underlying processes and their functional significance better, it would be important to conduct studies that investigate the consequences of beta‐rebound on subsequent processing, for instance in motor learning paradigms. If learning related feedback signals were somehow implied in the beta‐rebound (consistent with the idea of Donner and Siegel [2011] of this phenomenon playing a role for integrative purposes) one would expect variations in the beta‐rebound to inform subsequent processing. Top‐down processes are likely to influence neuronal excitation levels and may involve distinct neuromodulatory signals, including acetylcholinergic, so that the two perspectives offered here are distinct but non‐mutually exclusive.

In the present study, our findings already provide new information with regards to the PMBR which may help to disentangle functionally adaptive network processes such as top‐down inhibitory control, and basic cellular properties causing this phenomenon, leading to new hypotheses to be tested in future work. We show that, with greater force output an increase in PMBR amplitude is observed (Fig. 5) but there is no significant change in the duration of the response. This could suggest that a greater degree of inhibitory control is required following high force contraction in order to return to a pre‐contraction state. When peak force output is kept constant but the duration over which this force is attained is decreased (i.e. increasing RFD) PMBR duration decreases while amplitude increases (Fig. 6). We hypothesise that these two components of the rebound (amplitude and duration) might be affected by independent aspects of the task. We speculate that the variation in duration of the rebound might be related to the duration of the stimulus; i.e. longer stimulus durations will result in longer rebounds. Previous work [Stevenson et al., 2011] investigated the effect of increasing the duration of the motor stimulus (1 to 6 s) on beta responses and found increases in the integrated PMBR with increased stimulus duration up to 4 s. However, the paradigm design meant that as stimulus duration increased, the “rest period” decreased (minimum 4 s) in order to maintain a constant trial length. Therefore, any prolongation of PMBR may not have been fully captured, and more importantly, measures of baseline beta amplitude may have been collected during the latter stages of the PMBR, resulting in underestimation of the PMBR and overestimation of MRBD during the longer duration stimuli. Indeed short inter‐stimulus intervals are a consistent problem in studies of this type. Our current findings show that the duration of the PMBR can be in excess of 7 s. Therefore, when interpreting the changes in oscillatory power, both during and following stimulation, it is important to ensure that the electrophysiological response has fully returned to baseline before baseline itself is quantified.

The variation in amplitude of the rebound with RFD is less intuitive. Recall that in the RFD experiment, the peak force output is maintained for all RFDs, and only the rate at which that force is attained is varied. Our constant‐force experiment showed a clear increase in PMBR with force output and, if one believes the simplest hypothesis that PMBR amplitude is affected by force alone, then the finding of significantly modulated PMBR amplitude in the RFD experiment is counter intuitive. Although highly speculative, it is possible that this difference in amplitude may be driven by competing excitatory and inhibitory effects. We suggest that when contractions are sustained over a longer time period, the sensation of the handle in the subject's hand is retained following stimulus cessation, thereby causing a sensory aftereffect which may dampen the post‐stimulus rebound by evoking a concurrent stimulus for beta decrease. For example, it may be the case that, under a hypothesis that MRBD and PMBR have different neural generators, the sensory aftereffect would generate MRBD in sensory cortex, but the cessation of contraction would generate PMBR in motor cortex. The inherent spatial smoothness of the beamformer estimated time series would necessarily mix these two effects, so increased aftereffect induced MRBD could artifactually decrease the apparent PMBR. This dampening of post‐stimulus effects due to aftereffects has previously been reported for visual stimuli (static versus flashing checkerboards) in EEG alpha rebounds [Mullinger et al., 2015] and functional Magnetic Resonance Imaging (fMRI) post‐stimulus responses [Mullinger et al., 2015; Sadaghiani et al., 2009].

Our study has provided some evidence that the MRBD and PMBR originate via different neural generators. The idea that the MRBD and PMBR are fundamentally different processes is well established; for example, Donner and Siegel [2011] proposed that decreased beta amplitude is associated with local encoding processes, whereas increased post stimulus beta amplitude is associated with integrative processes over large networks. Indeed this idea was further explored by Liddle et al. [2016] and relates generally to evidence that beta oscillations may serve large‐scale communication between areas [Brookes et al., 2011; Hall et al., 2014a; Hipp et al., 2012; Kilavik et al., 2013]. Here, our observation that MRBD was related to neither force nor RFD, whereas PMBR was, adds support to these suggestions that MRBD and PMBR may be independent phenomena [Cassim et al., 2001; Feige et al., 1996; Jurkiewicz et al., 2006]. Additionally, we have shown that in one of our two experiments, a significant anterior shift in the spatial location of the PMBR compared to the MRBD is measurable. These results are in good agreement with several previous studies that have observed an anterior shift in the PMBR compared to the MRBD [Jurkiewicz et al., 2006; Salmelin et al., 1995; Stancák and Pfurtscheller, 1995] and such a spatial shift would be consistent with different cellular generators.

The data presented here have important implications for the interpretation of post‐stimulus undershoots measured in fMRI data [Buxton, 2012; van Zijl et al., 2012]. This part of the response was historically believed to be vascular in origin [Buxton et al., 1998] however there is a growing body of evidence that this response reflects neuronal activity [Mullinger et al., 2013; Sadaghiani et al., 2009; Shmuel et al., 2006]. Recent work has shown that the post‐stimulus fMRI response to a 10 s median nerve stimulus is correlated with the EEG alpha activity measured in a 10 s window post‐stimulation [Mullinger et al., 2013]. The work in this current study shows that, for long duration stimuli, the rebound will occur on this time scale, rather than 1–3 s as is often previously reported. This therefore provides potential new evidence for the link between electrophysiology and fMRI measures of post‐stimulus activity. Furthermore, previous studies have noted some changes in fMRI undershoot amplitude and/or duration with varying stimulus duration [Chen et al., 1998; Hu et al., 1997; Jin and Kim, 2008]. Conversely a separate study showed no significant effects of stimulus amplitude or duration on post‐stimulus undershoot responses [Chen and Pike, 2009] but in this study data had been grouped over visual and motor cortices which may have hidden the effects of interest in one sensory area. Further investigation is required to determine whether task‐specific modulations in the PMBR measured using MEG might translate to the post‐stimulus responses measured using fMRI and whether functional network processes or basic cellular processes account for these.

Finally, we note that here that, in the present study, it was our sole intention to investigate the dependence of beta band oscillatory phenomena on force output and RFD. However it is important to note that a number of other electrophysiological effects are observable in sensorimotor cortex [Cheyne, 2013] including, for example, the Bereitschaftspotential [Kornhuber and Deecke, 1965], the phase locked movement induced evoked response and the gamma band induced response. Previous work has suggested that the magnitude of these other movement‐related cortical potentials also modulates with both force and RFD [do Nascimento et al., 2005; Siemionow et al., 2000]. Future work should therefore aim to integrate these phase locked effects with measurements of oscillatory power.

## CONCLUSION

In this study, we have employed a carefully controlled isometric wrist flexion paradigm to isolate two fundamental movement parameters; force output, and the RFD. Our results show that the amplitude of MRBD is the same, regardless of changes in either force or RFD. In contrast, systematically changing the force output of a muscle contraction results in a monotonic modulation of PMBR, with a higher force outputs resulting in greater amplitudes of post‐stimulus response. Further, we found that increasing RFD generates significant increases in amplitude and decreases in duration of PMBR. These findings demonstrate that careful control of movement parameters can systematically change PMBR; further for temporally protracted movements (low RFD) the PMBR can be over 7 s in duration. This means accurate control of movement and judicious paradigm design are critical in future clinical and basic neuroscientific studies of sensorimotor cortex beta oscillations.

## Figures and Tables

**Figure A1:**
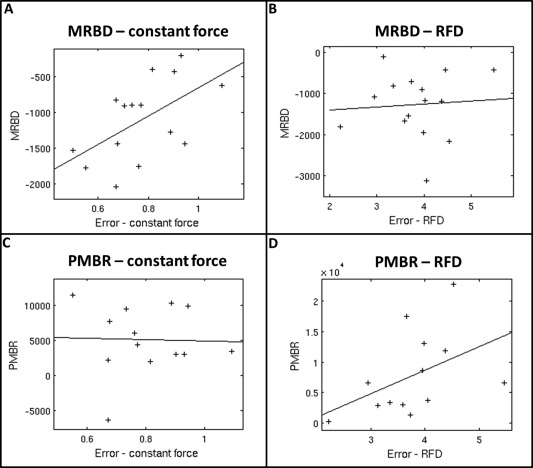
Correlation between subject accuracy and MRBD/PMBR: (**A**) MRBD vs mean error in force; constant‐force experiment. (**B**) MRBD vs mean error in force; RFD experiment. (**C**) PMBR vs mean error in force; constant‐force experiment. (**D**) PMBR vs mean error in force; RFD experiment.

## CORRELATION BETWEEN TASK ACCURACY AND MRBD/PMBR

The accuracy with which subjects were able to perform the isometric wrist flexion tasks was quantified based upon the error between measured and prescribed contraction force profiles. Overall error was defined as the absolute difference between the measured and prescribed force, averaged over time and trials. These values were collapsed across conditions for both the constant‐force and RFD experiments independently yielding a single measure relating to accuracy for each subject in both tasks. We then sought to probe any relationship between these accuracy metrics, and the associated beta modulation. Pearson correlation was calculated between MRBD/PMBR and accuracy (where PMBR/MRDB was collapsed across conditions in order to remove the effect of force/RFD). Significance was determined at 0.05, corrected for multiple comparisons across 4 measurements (2 tasks and 2 measurements (MRBD and PMBR)).

Results showed no significant relationship between accuracy and PMBR across subjects (Fig. 1C and A1D). A weak (r = 0.56; *P* = 0.03) trend was observed when assessing the relationship between MRBD and error for the constant‐force task (Fig. 1A); specifically, there was a tendency for subjects exhibiting a greater (more negative) MRBD to also exhibit higher accuracy. However, this observation was not reproduced for the RFD task (Fig. 1B) and was not significant following multiple comparison correction. However, it is possible that, given more subjects, such a result may prove robust and this should therefore form a topic for future investigation.
